# Impact on CO_2_/N_2_ and CO_2_/CH_4_ Separation Performance Using Cu-BTC with Supported Ionic Liquids-Based Mixed Matrix Membranes

**DOI:** 10.3390/membranes8040093

**Published:** 2018-10-11

**Authors:** Bernardo Monteiro, Ana R. Nabais, Maria H. Casimiro, Ana P. S. Martins, Rute O. Francisco, Luísa A. Neves, Cláudia C. L. Pereira

**Affiliations:** 1 Centro de Química Estrutural (CQE), Instituto Superior Técnico, Estrada Nacional 10, 2695-066 Bobadela, Portugal; bernardo.monteiro@ctn.tecnico.ulisboa.pt; 2 Centro de Ciências e Tecnologias Nucleares (C^2^TN), Instituto Superior Técnico, Estrada Nacional 10, 2695-066 Bobadela, Portugal; casimiro@ctn.tecnico.ulisboa.pt; 3 LAQV-REQUIMTE, Departamento de Química, Universidade Nova de Lisboa, 2829-516 Caparica, Portugal; a.nabais@campus.fct.unl.pt (A.R.N.); apd.martins@campus.fct.unl.pt) (A.P.S.M.); rr.francisco@campus.fct.unl.pt (R.O.F.)

**Keywords:** mixed matrix membranes, metal–organic frameworks, ionic liquids, gas separation, Cu_3_(BTC)_2_, IL@Cu_3_(BTC)_2_, IL@MOF

## Abstract

The efficient separation of gases has industrial, economic, and environmental importance. Here, we report the improvement in gas separation performance of a polyimide-based matrix (Matrimid^®^5218) filled with a Cu-based metal organic framework [MOF, Cu_3_(BTC)_2_] with two different ionic liquids (ILs) confined within the pores. The chosen ILs are commonly used in gas solubilization, 1-ethyl-3-methylimidazolium tetrafluoroborate ([EMIM][BF_4_]) and 1-Ethyl-3-methylimidazolium trifluoromethanesulfonate ([EMIM][OTf]), and the incorporation of the [EMIM][BF_4_]@Cu-BTC and [EMIM][OTf]@Cu-BTC composites in Matrimid^®^5218 proved to be an efficient strategy to improve the permeability and selectivity toward CO_2_/N_2_ and CO_2_/CH_4_ mixtures.

## 1. Introduction

Traditional methods used for gas separation, such as absorption using aqueous solution of amines, are being gradually substituted by adsorption-based technologies that are associated with a smaller ecological footprint. Different adsorbents have been studied for gas separation, including metal–organic frameworks (MOFs) [1,2]. MOFs have well-defined pore sizes, present a very high surface area, and have high gas adsorption capacities, and their characteristics (e.g., cavity size and functionalities) can be tuned by the selection of the most proper linker–metal pair. Several experimental and computational studies show that there are many MOFs that exhibit high adsorption selectivity towards CO_2_/CH_4_, CH_4_/H_2_, CO_2_/ N_2_, and CO_2_/H_2_ gas pairs [3].

ILs are liquid salts composed by an organic cation and an inorganic or organic anion. Their physical and chemical properties can be tuned by changing the cation or anion in their structure, similarly with the tunable linker–metal pair in MOFs. Additionally, in 2011, Yifei Chen et al. demonstrated, in a computational study, for the first time, that a composite formed by a metal–organic framework impregnated with an ionic liquid, IL@MOF, could be potentially useful for CO_2_ capture [4].

Composites formed by the incorporation of ILs within the pores of MOFs were recently reviewed in concern to their preparation and the challenges and opportunities of their applications [5,6].

Additionally, Vicent-Luna et al. reported the effect on CO_2_ separation of the Cu-BTC MOF when impregnated with different ILs based in the [EMIM]^+^ cation in combination with several anions [7]. This study of molecular simulations shows that the composite presents enhanced CO_2_ adsorption at low pressures, leading to higher adsorption selectivity for CO_2_ over CH_4_ and N_2_, when compared to the pristine Cu-BTC.

**Figure 1:**
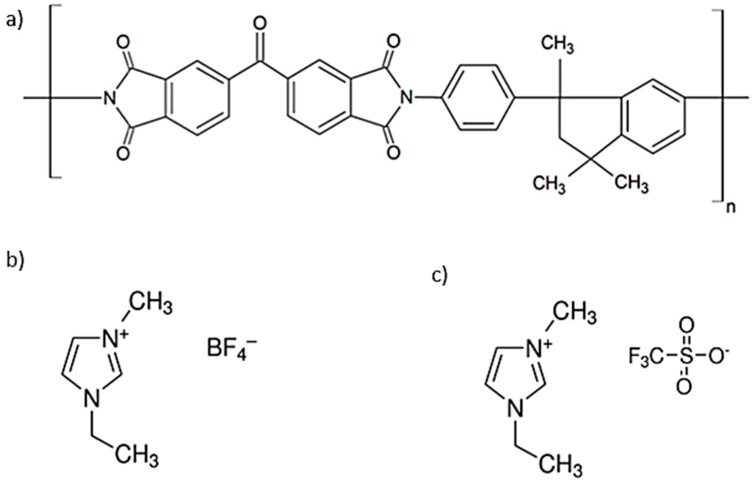
Schematic representation of the chemical structures of the (**a**) polymer Matrimid^®^5218, and the ILs (**b**) [EMIM][BF_4_] and (**c**) [EMIM][OTf].

The main objective of this work is the preparation of mixed matrix membranes (MMMs) formed by the dispersion of a MOF impregnated with task-specific ILs (MOF = Cu-BTC, ILs = [EMIM][BF_4_] or [EMIM][OTf]) within Matrimid^®^5218 forming polymeric structures (Figure 1) to overcome the empirical upper bound limits. To this end, we have recently reported how this methodology proved to be an efficient strategy to improve the membranes permeability and CO_2_/N_2_ ideal selectivity with a remarkable improvement in membrane flexibility and mechanical resistance [8]. MMMs are formed by the dispersion of filler particles in a polymeric matrix, and the properties of both the polymer and filler affect the separation performance. Several fillers for the preparation of mixed matrix membranes have been studied, such as zeolites, activated carbons, and MOFs. Overall, MMMs comprising an organic or inorganic filler proved to have high potential for gas separations [9].

The CO_2_/N_2_ and CO_2_/CH_4_ ideal selectivities at 30 °C of different composites of ILs incorporated in the Cu_3_(BTC)_2_, IL@Cu-BTC composites, dispersed in the polymeric membrane Matrimid^®^5218, was determined. The selection of the Cu-BTC MOF was based on previous reports that indicate high efficiency in CO_2_ sequestration. Due to their organic nature, MOFs are expected to show a better compatibility with polymers than other more traditional fillers (zeolites, silica and activated carbons), helping to avoid one of the most common problems of MMMs, the “sieve-in-a-cage-morphology”, i.e., the presence of defects or gaps between phases. In addition, to enhance the compatibility between MOF and polymer and improve the mechanical and transport properties, the use of ILs inside the porous structure of the MOF can be explored [8].

**Figure 2:**
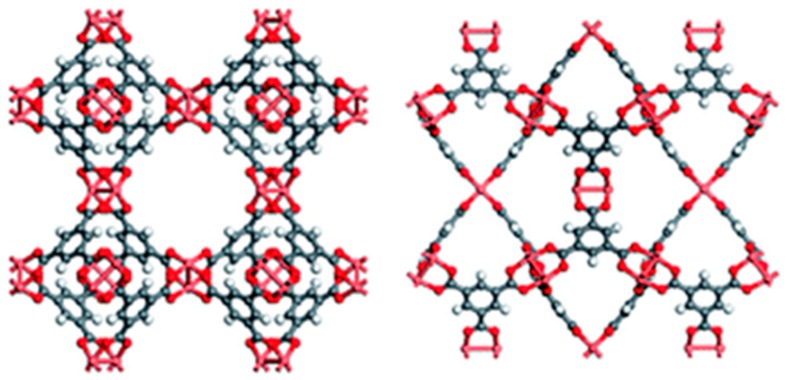
Cu-BTC metal–organic framework (MOF) viewed along two different axes to show the different pore structures. Adapted from a past study [11] with permission from The Royal Society of Chemistry.

The selection of the ILs was based on previous reports that indicate high efficiency and CO_2_ sequestration and solubilization [10]. 1-Ethyl-3-methylimidazolium tetrafluoroborate ([EMIM][BF_4_]) and 1-ethyl-3-methylimidazolium trifluoromethanesulfonate ([EMIM][OTf]) (Figure 1) were selected as ILs for combination with Cu_3_(BTC)_2_ MOF, also known as HKUST-1 (Figure 2). The porous framework of Cu-BTC has the formula unit Cu_3_(BTC)_2_, with the organic benzene-1,3,5-tricarboxylate ligand acting as linker of dicopper tetracarboxylate paddlewheel secondary building units. Additionally, it is noteworthy that the coordinatively unsaturated Cu^2+^ sites can interact with free electrons of small molecules [11,12].

## 2. Materials and Methods

### 2.1. Materials

Reagent grade chemicals were obtained from Sigma-Aldrich (St. Louis, MO, USA) and used without further purification. [EMIM][OTf] and [EMIM][BF_4_] (Iolitec) were used as supplied, and deionized water was processed by Diwer Technologies water max w2 equipment (Weger Walter GmbH, Zona Artigianale, Italy).

### 2.2. Synthesis

Cu_3_(BTC)_2_, also referred as Cu-BTC, was synthesized according to previously reported methods [13].

### 2.3. Preparation of IL@MOF

Before the impregnation, 150 mg of Cu-BTC was activated by heating at 100 °C with simultaneous reduced pressure for 1 h. Then, maintaining the reduced pressure, the selected IL was added with a syringe until all the Cu-BTC got covered by the IL. These solutions were sonicated for 4 h, and then left under magnetic stirring for 24 h. They were then mixed with the Matrimid^®^5218 solutions and further agitated for 1 h before pouring them into petri dishes. The final MMMs were obtained by slow evaporation of the solvent in desiccators.

### 2.4. Membranes Preparation

Different membranes were prepared, namely Matrimid^®^5218, mixed matrix membranes (MMMs) composed of Matrimid^®^5218 and the metal organic framework Cu_3_BTC_2_, and mixed matrix membranes with a low percentage (10% *w/w*) of IL@MOFs composites (MMMs-ILs@MOFs). All membranes were prepared by the solvent evaporation method. Solutions of Matrimid^®^5218 were prepared by dissolving 0.5 g Matrimid^®^5218 in 4.5 mL of dichloromethane. The additive solutions (MOF and IL@MOFs) were prepared in separate vials in dichloromethane, where the additive loading was between 10% and 30%, and 10% (*w/w*), respectively. The solutions were then sonicated for 4 h and agitated for 24 h separately on magnetic stirrers. They were then mixed and agitated for 1 h before pouring them into petri dish and kept in desiccators for slow evaporation of the solvent.

### 2.5. Pure Gas Permeation Experiments

The pure N_2_, CH_4_, and CO_2_ permeation experiments were carried out using a gas permeation setup developed previously [14]. The experimental apparatus is composed of a stainless-steel cell with two identical compartments separated by the membrane. The experimental setup was placed in a thermostatic water bath (Julabo GmBH ED, Seelbach, Germany) at a constant temperature of 30 °C. Each experiment started by pressurizing both compartments with the pure gas (N_2_, CH_4_ or CO_2_), and a driving force of 0.7 bar of relative pressure, between both compartments, was established. The pressure evolution in both compartments was measured by using two pressure transducers (Druck PCDR 910, models 99166 and 991675, UK). The permeability of a pure gas through the membrane was calculated according to the equation:

$$\frac{1}{\beta }\ln\left(\frac{p_{feed0}-p_{perm0}}{p_{feed}-p_{perm}}\right)=P\frac{t}{l}$$

where *p_feed_* and *p_perm_* are the pressures (bar) in the feed and permeate compartments, respectively, *P* is the membrane permeability (m^2^·s^−1^), *t* is the time (s), and *l* is the membrane thickness (m). *β* (m^−1^) is a geometric parameter, characteristic of the cell geometry and is given by

$$\beta =A\left(\frac{1}{V_{feed}}+\frac{1}{V_{perm}}\right)$$

where *A* is the membrane area (m^2^) and *V_feed_* and *V_perm_* are the volumes (m^3^) of the feed and permeate compartments, respectively [15]. The gas permeability is obtained from the slope when representing *1/β ln∆p*_0_*/∆p* as a function of *t/l*.

The ideal gas selectivity was calculated by

αAB=PAPB

## 3. Results

### 3.1. Composite Characterization

**Figure 3:**
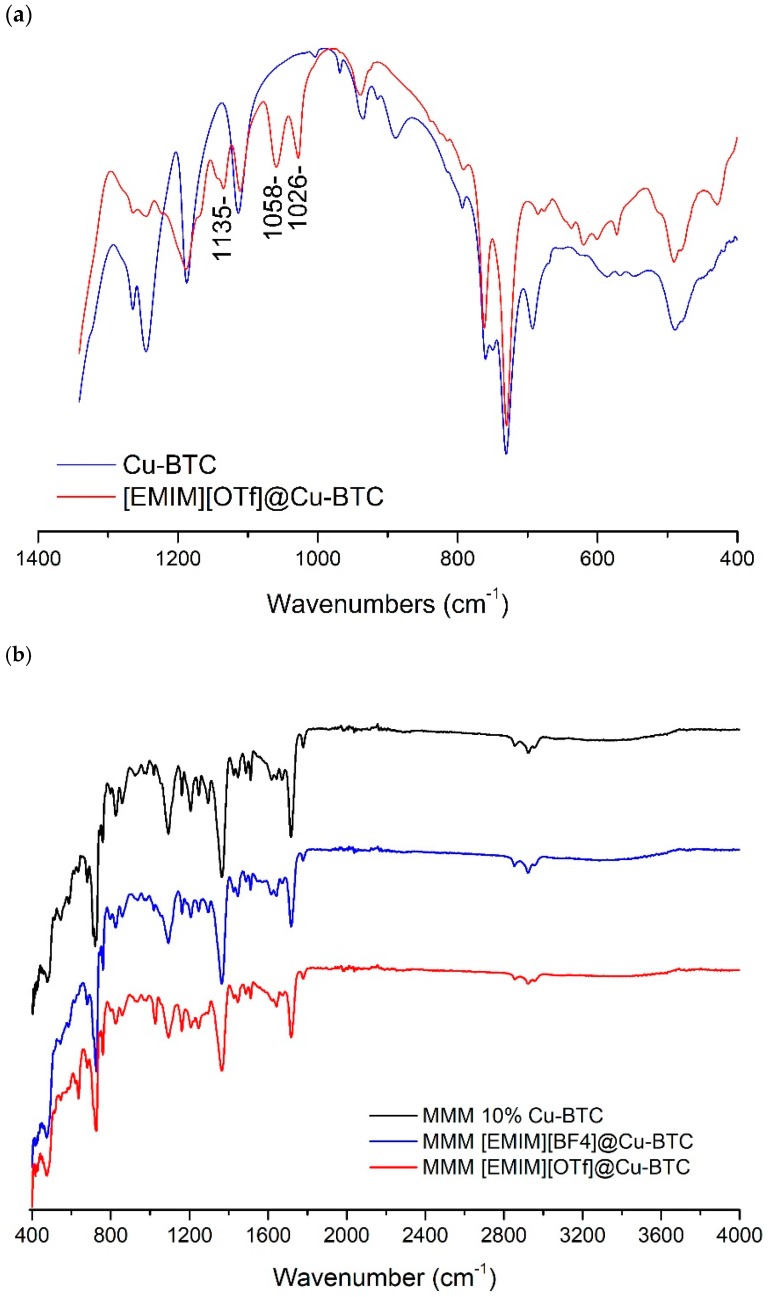
(**a**) Partial infrared spectra of Cu-BTC and EMIMOTf@Cu-BTC; (**b**) FTIR spectra of the mixed matrix membranes prepared in this work.

Infrared absorption (IR) measurements have been conducted to confirm the existence of ILs inside the pores of Cu-BTC. The FT-IR spectra of [EMIM][OTf]@Cu-BTC and pure IL (Figure 3) show IR bands around 1026, 1058, and 1135 cm^−1^, which were exclusively found in the supported ionic liquid and are due to the SO_3_ vibration of the OTf^−^ moieties (1026 cm^−1^) and CF_3_ asymmetric vibrations, respectively [16]. In the case of the [EMIM][BF_4_]@Cu-BTC composite, the presence of the IL is discernible by the appearance of the band at 1168 cm^−1^ ascribed to the C–H in-plane vibration of the EMIM^+^ cation and another band at 1037 cm^−1^ due to the B–F vibrations in the BF_4_^−^ anion (Figure S1 in Supplementary Materials).

From the FTIR spectrum of the IL@Cu(BTC)-based MMMs (Figure 3b), it is possible to see that there are some interactions being established between the MOF and the IL in the membrane. For both IL@Cu(BTC) MMMs, there is a slight peak location shift and change in intensity, compared to the membrane with 10% Cu(BTC), at around 1100 cm^−1^. The same behavior can be observed at wavenumbers between 1600 and 1800 cm^−1^. Usually, any change in the peak location or in peak intensity is due to the formation of chemical and physical interactions in the membrane that can impact on the membranes’ separation performance.

In this particular case, these interactions between the materials resulted in membranes with higher CO_2_/N_2_ selectivity, compared to the Matrimid^®^ and Matrimid^®^_Cu(BTC) membranes.

**Figure 4:**
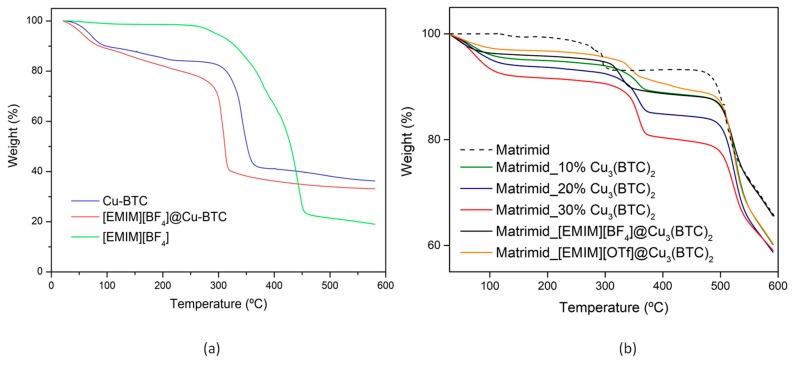
Thermogravimetric analysis (**a**) of [EMIM][OTf] (green line), Cu-BTC (blue line), and [EMIM][OTf]@Cu-BTC (red line) in the range 20 to 600 °C. The mixed matrix membranes (MMMs) (**b**).

Thermogravimetric analysis (TGA) of the samples showed that the composites samples have more weight loss than pristine Cu-BTC upon heating to 600 °C in flowing N_2_ due to the presence of the organic ILs. As can be seen in Figure 4a, for the [EMIM][OTf] series, the Cu-BTC precursor mostly decomposes in the range 300 to 350 °C and the IL starts to decompose only around 350 °C. So, as expected, the decomposition of the [EMIM][OTf]@Cu-BTC composite, starts around 300 °C due to the structural decomposition of the Cu-BTC (weight loss around 60% at 375 °C), compared to 3% of neat [EMIM][OTf] at the same temperature, and continues to decompose after 350 °C following the decomposition of the IL. Above this temperature and until 470 °C, the weight loss is higher for IL@MOF (8%) when compared with Cu-BTC (3%). This is the expected, and is due to the difference corresponding to the IL decomposition at 470 °C. In the case of the [EMIM][BF_4_], it presents the opposite case, i.e., the IL starts to decompose at lower temperatures than the MOF precursor and so the [EMIM][BF_4_]@Cu-BTC composite starts it decomposition before the pristine Cu-BTC and then accompanies the MOF decomposition (Figure S2 in Supplementary Materials).

The TGA profiles show that, for all Cu(BTC)-containing MMMs there is an initial weight loss, possibly related with the evaporation of residual trapped solvent. For the MMMs with different Cu(BTC) loadings (10, 20, and 30% (*w/w*)), the weight loss is more significant with increasing MOF content, however, the thermal decomposition temperature (Td) of the MMMs is not significantly affected. The Td of the Cu(BTC)-based MMMs is higher than that of the pure polymeric membrane, which translates to higher thermal stability, possibly due to the high thermal stability of the incorporated MOF.

For the IL@Cu(BTC)-based MMMs, it can be seen that the TGA profiles are very similar, with Matrimid^®^_[EMIM][OTf]@Cu(BTC) presenting the highest thermal stability.

Overall, the incorporation of Cu(BTC) and IL@Cu(BTC) particles in the polymeric matrix resulted in an improvement of the thermal properties of the membranes.

**Figure 5:**
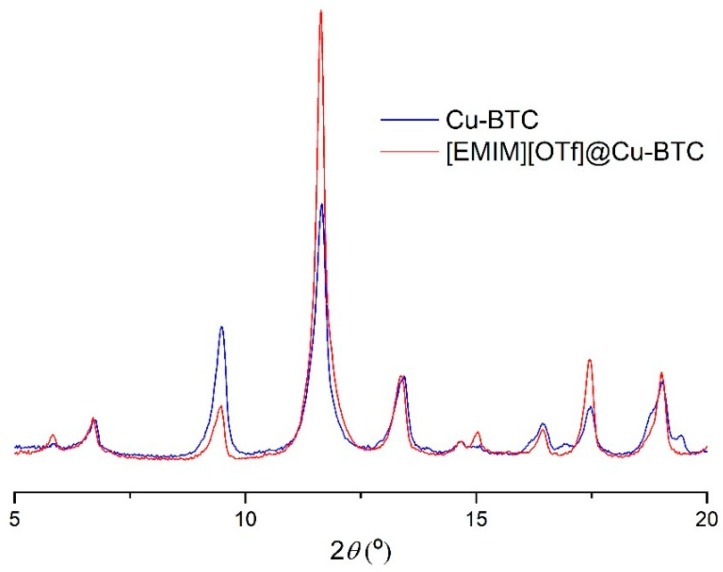
Powder XRD pattern for the precursor Cu-BTC (blue line) and the [EMIM][OTf]@Cu-BTC composite (red line).

**Figure 6:**
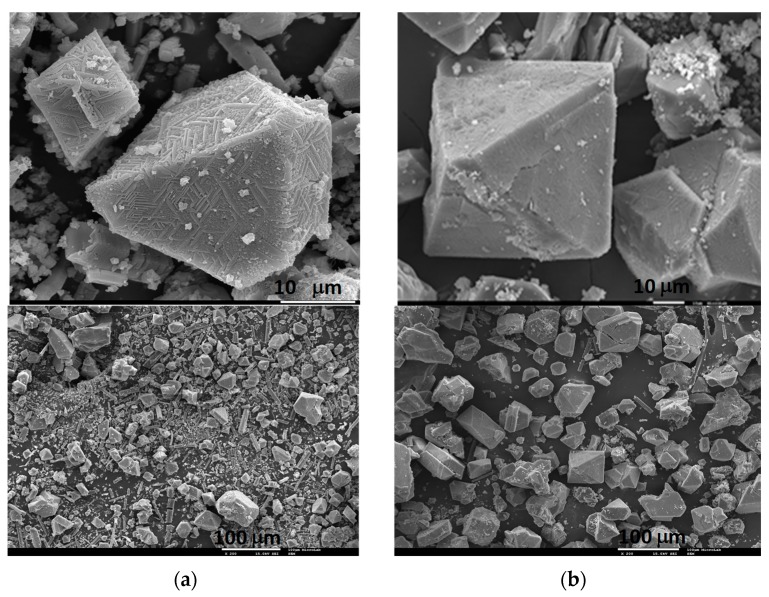
SEM images of (**a**) Cu-BTC and (**b**) [EMIM][OTf]@Cu-BTC composite.

The powder X-ray diffraction studies of the IL-incorporated samples shows that the framework of the Cu-BTC precursor does not change with the incorporation of the ILs (Figure 5 and Figure S3) which is consistent with the SEM images (provided as Figure 6 and Figure S4). As a result, it can be inferred that the structures did not show any deformation or loss of crystallinity after impregnation.

Moreover, after incorporation of near 10 wt % ILs, according to the elemental analysis results, inside the MOF, the ILs@Cu-BTC composites remained as dry powders, further indicating that the IL molecules are not at the external surface but inside the pores.

### 3.2. Gas Permeation Studies

**Figure 7:**
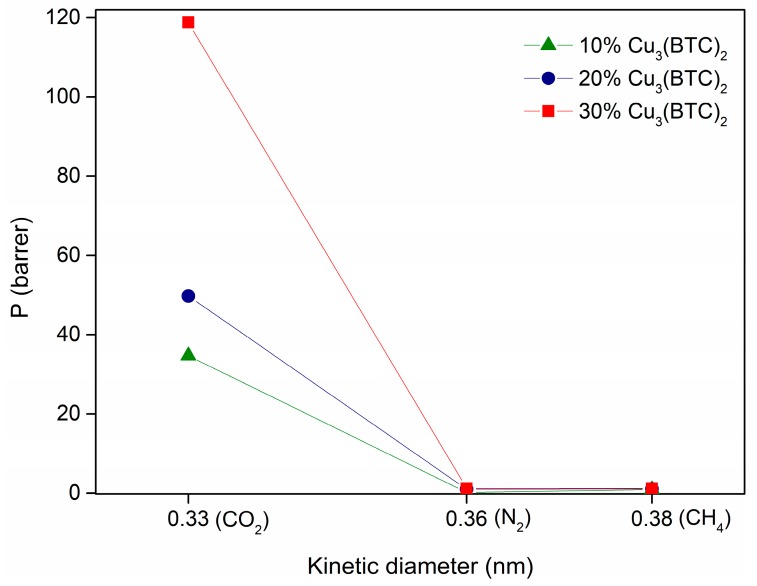
Evolution of gas permeability as a function of the gas kinetic diameter for MMMs prepared with different loadings of Cu-BTC (between 10 and 30% *w/w*).

In a first study, mixed matrix membranes were prepared using Matrimid@5218 with different loadings (of 10, 20, and 30% (*w/w*)) of Cu-BTC MOF. It has been observed (Figure 7) that with an increase in MOF loading, for all the gases studied, an increase in gas permeability was obtained. These results agree with those available in the literature [17,18,19]. The incorporation and increasing MOF content in the membrane provides an extra pore network, due to the high porosity of Cu-BTC. Moreover, it is also possible that the addition of MOF particles in the polymeric matrix, results in an increase of the d-spacing, which results in a higher interchain distance and, consequently in an increased available free volume, as observed in previous studies [20]. This contributes to a higher diffusion of the gases through the membrane and, consequently, in the observed improvement in permeability.

For the same MOF loading, gas permeability increases in the order N_2_ < CH_4_ < CO_2_. Due to its quadrupole moment, CO_2_ has a higher affinity for the Matrimid@5218, which results in a higher solubility. Moreover, the favored CO_2_ transport may also be related with a higher diffusion of this gas through the membrane, due to its smaller kinetic diameter (0.33 nm), compared to the other gases.

**Figure 8:**
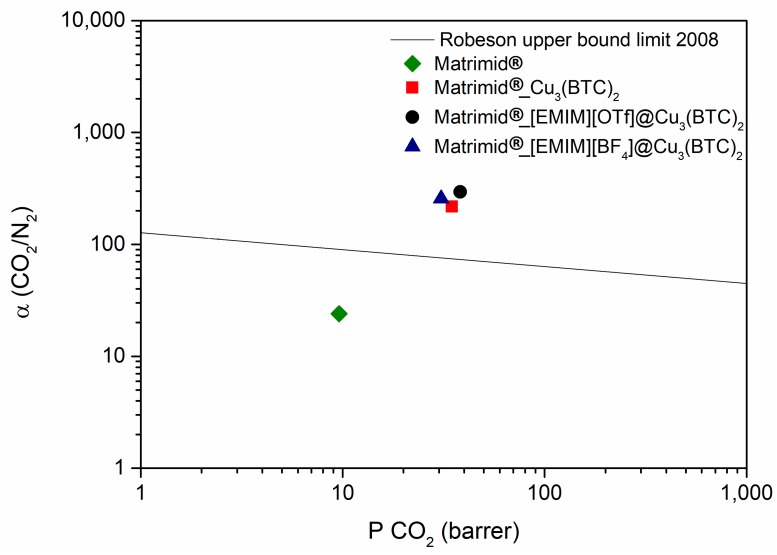
CO_2_/N_2_ ideal selectivity as a function of CO_2_ permeability for the MMMs prepared. Comparison with the 2008 Robeson upper bound for CO_2_/N_2_ separation [21].

**Figure 9:**
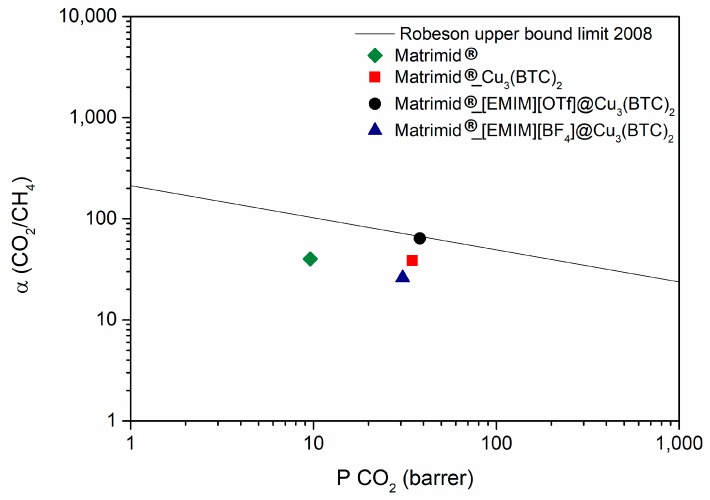
CO_2_/CH_4_ ideal selectivity as a function of CO_2_ permeability for the MMMs prepared. Comparison with the 2008 Robeson upper bound for CO_2_/CH_4_ separation [21].

In Figure 8 and Figure 9 are represented the results obtained for the CO_2_ permeability and CO_2_/N_2_ and CO_2_/CH_4_ ideal selectivity, respectively, for the IL@Cu-BTC composite mixed matrix membranes prepared in this work, IL@Cu-BTC@Matrimid@5218. It is possible to see that, in both cases, there is an improvement in the CO_2_ permeability and ideal selectivity of Cu-BTC and IL@Cu-BTC-filled MMMs when compared with Matrimid@5218. For both separations, the highest CO_2_ permeability was achieved for the [EMIM][OTf]@Cu-BTC@Matrimid, and this may be because this IL presents a higher solubility towards CO_2_ compared to [EMIM][BF_4_].

For the CO_2_/N_2_ separation (Figure 8), all prepared MMMs were able to surpass the permeability/selectivity trade-off limit. Moreover, the IL@Cu-BTC@Matrimid MMMs show a slightly improvement in ideal selectivity, over the membrane prepared only with the MOF. It is known that ILs have much higher affinity for CO_2_ compared with other gases, which means that the introduction of ILs in the pores of the Cu-BTC may improve the adsorption selectivity in the membranes. Moreover, in the case of the IL@MOF composites, the MOF porous cavity is partially occupied by the IL, and since CO_2_ (0.33 nm) has a smaller kinetic diameter compared to N_2_ (0.36 nm), the diffusion of the smaller gas through the membrane is higher, which results in an improvement of the CO_2_/N_2_ ideal selectivity.

Regarding the CO_2_/CH_4_ separation (Figure 9), when comparing the Cu-BTC@Matrimid and [EMIM][BF_4_]@Cu-BTC@Matrimid membranes, it is possible to see that the introduction of the IL in the MOF cavities resulted in a slight decrease of the CO_2_ permeability. This may be the result of a decrease in the gas diffusivity, because the IL partially occupies the MOF cavity. As for the [EMIM][OTf]@Cu-BTC@Matrimid membrane, this behavior was not observed possibly because the high CO_2_ solubility of this IL was able to surpass an eventual decrease in diffusivity, as previously explained.

## 4. Conclusions

Summarizing, for the CO_2_/N_2_ separation, all prepared MMMs were able to surpass the permeability/selectivity trade-off limit.

In terms of CO_2_/CH_4_ ideal selectivity, the Cu-BTC@Matrimid and [EMIM][OTf]@Cu-BTC@Matrimid membranes show an improvement, compared to the polymeric membrane. On the other hand, the introduction of [EMIM][BF_4_] in the pores of Cu-BTC resulted in a slight decrease in CO_2_/CH_4_ ideal selectivity, compared to the Cu-BTC@Matrimid membrane. In this case, [EMIM][BF_4_] shows the highest affinity toward CH_4_, even higher than that of CO_2_, which causes the ideal selectivity to decrease [19].

## Supplementary Materials

The following are available online at http://www.mdpi.com/2077-0375/8/4/93/s1. Figure S1: FT-IR spectra of the Cu-BTC precursor (blue line) and the composite [EMIM][BF_4_]@Cu-BTC (red line) collected in the 400–4000 cm^−1^ range. Figure S2: Thermogravimetric analysis of [EMIM][BF_4_] (green line), Cu-BTC (blue line) and [EMIM][BF_4_]@Cu-BTC (red line) in the range 20–600 °C. Figure S3: Powder XRD pattern for the precursor Cu-BTC (blue line) and the [EMIM][BF_4_]@Cu-BTC composite (red line). Figure S4: SEM images of the [EMIM][BF_4_]@Cu-BTC composite.
