# Supplementary material for: Impact on CO2/N2 and CO2/CH4 Separation Performance Using Cu-BTC with Supported Ionic Liquids-Based Mixed Matrix Membranes
Source: Membranes (Basel). 2018 Oct 11;8(4):93. doi: 10.3390/membranes8040093 (PMC6316195; doi:10.3390/membranes8040093)
Supplement: Supplementary file 1 [file membranes-08-00093-s001.pdf]

membranes

Supplementary Materials: Impact on CO_2/N_2 and CO_2/CH_4 Separation Performance Using Cu-BTC with Supported Ionic Liquids-Based Mixed Matrix Membranes

Bernardo Monteiro [1,2], Ana R. Nabais [3], Maria H. Casimiro [2], Ana P. S. Martins [3], Rute O. Francisco [3], Luisa A. Neves [3,*] and Cláudia C. L. Pereira [3,*]

[1] Centro de Química Estrutural (CQE), Instituto Superior Técnico, Estrada Nacional 10, 2695-066 Bobadela, Portugal; bernardo.monteiro@ctn.tecnico.ulisboa.pt
[2] Centro de Ciências e Tecnologias Nucleares (C[2]TN), Instituto Superior Técnico, Estrada Nacional 10, 2695-066 Bobadela, Portugal; casimiro@ctn.tecnico.ulisboa.pt
[3] LAQV-REQUIMTE, Departamento de Química, Universidade Nova de Lisboa, 2829-516 Caparica, Portugal; a.nabais@campus.fct.unl.pt (A.R.N.); apd.martins@campus.fct.unl.pt) (A.P.S.M.); rr.francisco@campus.fct.unl.pt (R.O.F.)
* Correspondence: luisa.neves@fct.unl.pt or lan11892@fct.unl.pt (L.A.N.); ccl.pereira@fct.unl.pt (C.C.L.P.)

[EMIM][BF$_4$] series

Infrared spectroscopy

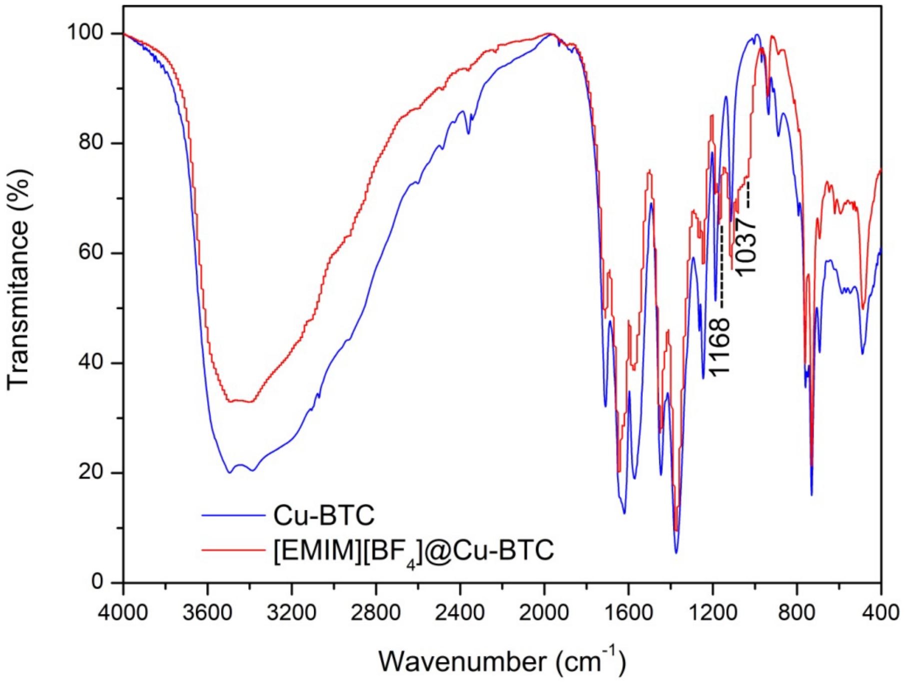

Figure S1. FT-IR spectra of the Cu-BTC precursor (blue line) and the composite [EMIM][BF$_4$]@Cu-BTC (red line) collected in the 400 – 4000 cm^{-1} range.

Thermogravimetry

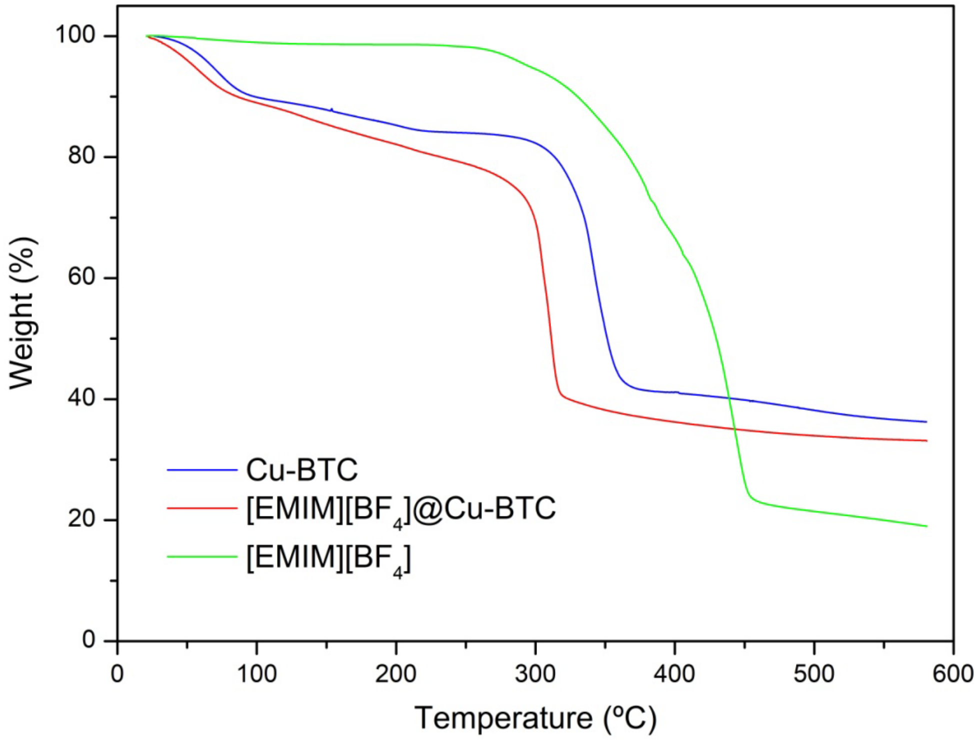

Figure S2. Thermogravimetric analysis of [EMIM][BF_4] (green line), Cu-BTC (blue line) and [EMIM][BF_4]@Cu-BTC (red line) in the range 20–600 °C.

Powder X-ray diffraction

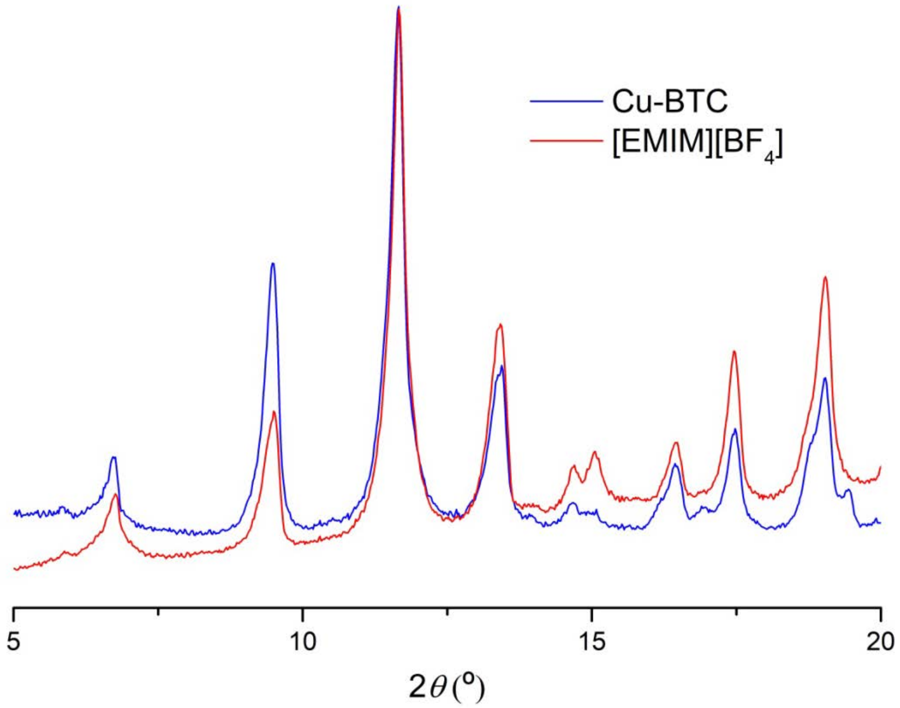

Figure S3. Powder XRD pattern for the precursor Cu-BTC (blue line) and the [EMIM][BF_4]@Cu-BTC composite (red line).

Scanning Electron Microscopy (SEM)

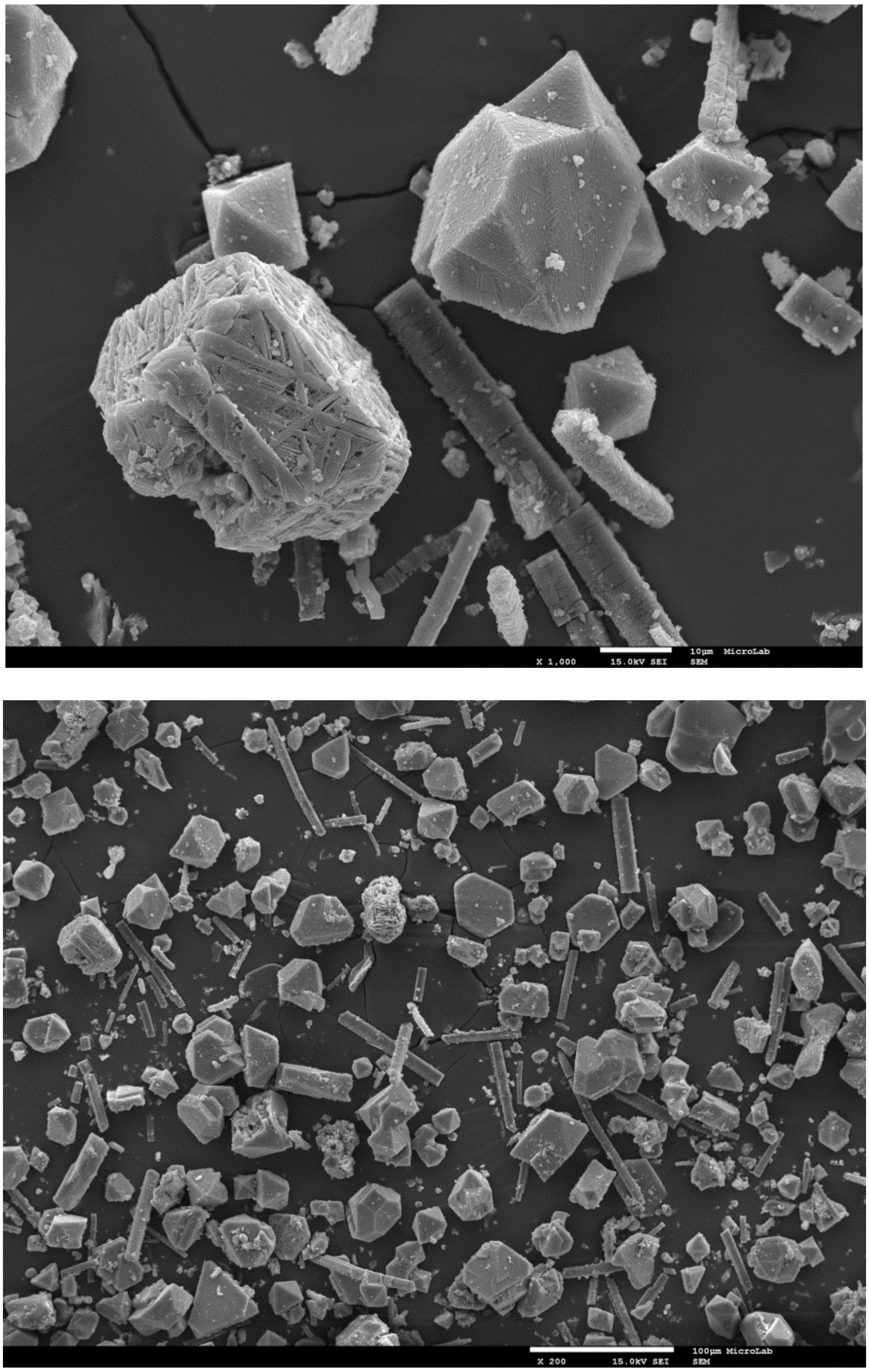

Figure S4. SEM images of the [EMIM][BF_4]@Cu-BTC composite.

© 2018 by the authors. Submitted for possible open access publication under the terms and conditions of the Creative Commons Attribution (CC BY) license (http://creativecommons.org/licenses/by/4.0/).
